# Pirfenidone in Skin Fibrosis and Scarring: From Bench Insights to Clinical Data

**DOI:** 10.3390/medsci13030148

**Published:** 2025-08-20

**Authors:** Kelson Knighton, Asis Babun, James Turney, Brehyn Evans, Inder Sehgal

**Affiliations:** Department of Biomedical Sciences, Rocky Vista University, Ivins, UT 84738, USA; asis.babun@ut.rvu.edu (A.B.); james.turney@ut.rvu.edu (J.T.); brehyn.evans@ut.rvu.edu (B.E.);

**Keywords:** pirfenidone, scar, hypertrophic scar, keloid

## Abstract

Pirfenidone (PFD) is a pyridine-like compound most well-known for its use in idiopathic pulmonary fibrosis (IPF). However, its broad anti-inflammatory and anti-fibrotic actions make PFD a candidate for other scarring diseases. This review examines the use of PFD for dermatologic conditions. The literature supports the potential for PFD to decrease scarring in a variety of skin conditions. Both oral and topical preparations have been shown to be effective at aiding skin healing. Early clinical evidence demonstrates significant improvements in hypertrophic burn scars, reduction in fibrosis in localized scleroderma, and accelerated epithelialization of skin graft donor sites. These clinical outcomes are supported by PFD’s modulation of the transforming growth factor-beta (TGF-β) pathway, which plays a central role in skin fibrosis and scarring. Evidence in this review suggests topical PFD can be used to fill a clinical need for local anti-fibrotic therapies.

## 1. Introduction

Fibrosis, characterized by the excessive accumulation of extracellular matrix components, leads to tissue scarring and impaired organ function. This process represents a significant global health issue, implicated in as many as 45% of all deaths in the industrialized world [1,2]. Skin fibrosis, often resulting from trauma, surgery, or burns, can significantly impact a patient’s quality of life by causing disfigurement and functional impairments. Scarring of the skin is also very common, with nearly half of adults worldwide reporting at least one scar [3]. Pathological scarring remains a persistent therapeutic challenge, with no single treatment universally accepted as a gold standard [4,5]. This creates a critical need for novel anti-fibrotic strategies to improve patient outcomes.

Pirfenidone (5-methyl-1-phenyl-1H-pyridin-2-one) was first identified in the 1990s as an anti-fibrotic agent [6,7]. It was quickly shown to ameliorate fibrosis in numerous animal models for a variety of diseases [7,8,9,10,11,12,13,14]. Oral pirfenidone (PFD) is rapidly absorbed, extensively metabolized by the liver, and has a short elimination half-life [15,16,17]. Its mechanism is primarily attributed to inhibiting transforming growth factor-beta (TGF-β), a key regulator of fibrotic processes [18,19,20].

Clinical trials for lung fibrosis led to the drug’s approval to treat idiopathic pulmonary fibrosis (IPF) in Europe, Japan, and the United States of America [21,22,23]. Since then, there has been interest in re-purposing PFD for other fibrotic diseases [24]. Although PFD is administered orally in the U.S. and Canada, other countries have explored its topical application to manage skin fibrosis, particularly in the treatment of scars [24,25]. Topical application is advantageous as it avoids the considerable gastrointestinal side effects reported with oral PFD [26,27].

This review examines the efficacy of pirfenidone to prevent scarring and its potential in related dermatologic conditions. Previous articles have reviewed pirfenidone’s use in other conditions [20,22] or have provided a broad overview of the drug [24]. The aim of this review is to synthesize the existing literature on both topical and systemic pirfenidone for the treatment of skin fibrosis and scarring. Specifically, we evaluate the mechanistic evidence, clinical efficacy, and safety profile of pirfenidone to clarify its potential role in dermatology. The evidence for both topical and systemic use is discussed. This content is useful to those seeking innovative, low-risk, and effective local options for scarring and other fibrotic skin conditions.

## 2. Materials and Methods

**Figure 1:**
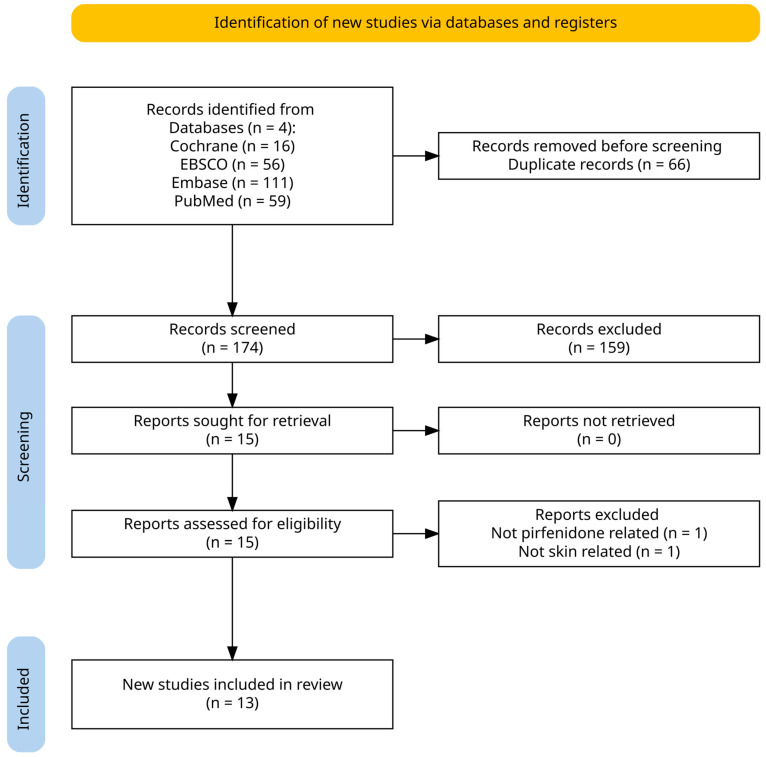
Identification of studies via databases.

Consistent with best practices, a search was performed in Cochrane Library, EBSCO, Embase, and PubMed. Keywords and MeSH terms including “pirfenidone”, “skin”, and “scar” were used to identify records. An a priori eligibility framework and screening plan were established before the search commenced. Articles were included when they examined the use of pirfenidone for any cutaneous condition regardless of participant age, geographic location, study design, setting, or peer-review status. Both human and non-human studies were included. No limits on date, language, study design, or publication type were applied. A total of 174 records were identified after the removal of duplicates. Search data was then extracted into a standard Excel spreadsheet for review by the authors. Data from all searches were transferred to a standardized Excel sheet for analysis. Knighton and Turney independently screened the titles and abstracts, selecting 15 papers for full-text evaluation. The ensuing full-text review was performed separately by Knighton, Babun, and Evans; any discrepancies were settled through consensus discussion. Two records were excluded for irrelevance, leaving 13 studies for qualitative synthesis. No formal risk-of-bias appraisal was undertaken. Figure 1 is a PRISMA diagram of the search and screening process.

## 3. Results

### 3.1. Mechanism

The mechanisms of PFD may vary depending on tissue location; therefore, this review will focus on evidence collected from models of skin disease. PFD appears to be well absorbed into the skin [28,29], and most studies from our search used topical formulations including gel, ointment, and patch [30,31,32,33,34,35,36]. Since topical PFD application avoids first-pass metabolism [24], monitoring liver enzymes may not be necessary [37,38]. A number of different topical delivery mechanisms have been explored [28,29,30,31,33,35,36], but a lack of direct comparisons between methods obscures identification of an optimal medium. Trials with topical PFD have shown efficacious delivery as gels [32,36], ointments [28], and in a variety of unique dressings [29,30,31,33,35]. Chung et al. compared a 15% pirfenidone soft skin adhesive patch to an 8% pirfenidone gel in an ex vivo human skin model. They found the patch to have greater flux and total permeation over 3 days [29]. Dorati et al. in a dose study found that a 6.5% PFD ointment would decrease TNF-α and IL-12 expression in a mouse burn model, but 1% and 3.5% ointments had no effect [28]. More work is needed to understand the optimal delivery method and dosing for PFD; however, more commercial forms are gels at 8% [37].

Once PFD permeates into live cell membranes, the small, hydrophobic molecule likely diffuses into the cell without the use of a receptor [20]. Inside the cell, PFD suppresses fibrosis and inflammation through a variety of mechanisms. Putative mechanisms include scavenging reactive oxygen species [39], immune regulation [19,40,41,42], and inhibiting fibroblast differentiation [18,43].

The best characterized mechanism of action for pirfenidone is through attenuation of the transforming growth factor beta (TGF-β) pathway. TGF-β has been shown to be central in the scarring process for skin [44]. PFD treatment decreases TGF-β mRNA expression in keratinocytes and dermal fibroblasts [45,46,47,48]. It also works further downstream in the TGF-β/SMAD pathway by decreasing phosphorylation in SMAD3 and p38 MAPK proteins [43,47,49]. Much of the existing dermatologic research has focused on the classical TGF-β pathway, and only recently has there been an investigation into how PFD inhibits fibroblast proliferation through dampening of the Wnt/GSK-3β/β-catenin cascade [50]. The cumulative effect is less collagen deposition, less scar contraction, and attenuated scar formation [35,43,45,48,49,51].

PFD has wide-ranging systematic effects, including modulating the effects of the immune system [20,24]. While it is not known exactly how PFD changes immune cell function in skin, it decreases macrophage infiltration of burn wounds and downregulates a number of pro-inflammatory cytokines [28,30,49]. This suggests that pirfenidone can be useful during the inflammation stage of wound healing by calming an overactive immune response. A shortened inflammation stage could explain the quickened healing times shown in animals [30,31,35] and humans [52,53]. PFD has also shown positive effects in the later stages of wound healing. During the proliferative phase it decreased collagen I/III deposition [30,47,51] and inhibited the epithelial-to-mesenchymal transition [46,54]. Lastly, PFD can enhance remodeling through upregulating the expression of metalloproteases [29,48] and inhibiting wound contraction [33,45,49].

### 3.2. Clinical Evidence

To date, four clinical trials have studied PFD use for skin conditions [32,36,52,53]. All studies took place in Mexico, where an 8% pirfenidone gel (Kitoscell) is commercially available and used for scars [37,55]. The gel has been used as prophylaxis for scarring and decreasing recovery time following reconstructive surgery [56]. Despite anecdotal reports of its widespread use, few patients have been enrolled in studies about pirfenidone for skin conditions. A summary of the clinical evidence is presented in Table 1.

#### 3.2.1. A Controlled Clinical Trial with Pirfenidone in the Treatment of Pathological Skin Scarring Caused by Burns in Pediatric Patients

This prospective clinical trial assessed the safety and efficacy of 8% topical pirfenidone gel, applied three times a day, in reducing hypertrophic scars secondary to burn injuries in pediatric patients [32]. The study included 33 pediatric patients treated with pirfenidone gel and 30 patients treated with standard pressure therapy as a control group. The results showed that the pirfenidone group demonstrated a statistically significant monthly reduction in Vancouver Scar Scale (VSS) scores [32], with an overall improvement of 34% in scar characteristics compared to the control group after 6 months. Specifically, 27% of the patients in the pirfenidone group achieved a VSS reduction of >55%, and 67% saw a moderate improvement of 30–45%.

The study concluded that topical pirfenidone gel significantly improves the appearance and structure of hypertrophic burn scars in pediatric patients compared to standard pressure therapy, without notable side effects. Patient’s age, location of scars, and time since injury were not reported.

#### 3.2.2. Pirfenidone Gel in Patients with Localized Scleroderma: A Phase II Study

This phase II study evaluated the safety and efficacy of 8% topical pirfenidone gel in reducing fibrosis and skin hardness in localized scleroderma lesions [36]. The study included 12 patients with histologically confirmed active localized scleroderma who applied pirfenidone gel three times daily over six months. The primary outcomes included changes in skin severity using the modified Localized Scleroderma Skin Severity Index (mLoSSI) [36,57], cutaneous induration via durometry, and histopathological alterations.

The study demonstrated significant reductions in mLoSSI scores, with an average decrease of 5.83 ± 4.80 to 0.83 ± 1.75 at 6 months (*p* = 0.002). Histopathological analysis showed improvements in epidermal atrophy, dermal infiltration, and reticular dermis fibrosis, indicating a reduction in collagen density and fibrosis. The treatment was well-tolerated, with minimal side effects and no systemic toxicity. The study’s small sample size and open-label design limit its generalizability; however, the findings suggest that pirfenidone could be an effective and safe addition to localized scleroderma management protocols.

#### 3.2.3. Pirfenidone Increases the Epithelialization Rate of Skin Graft Donor Sites

This randomized, controlled, prospective trial evaluated the efficacy of 8% pirfenidone gel in enhancing epithelialization of skin graft donor sites [52]. The study involved 28 participants, with 19 in the treatment group receiving pirfenidone gel and non-adherent gauze, and 9 in the control group treated with the gauze alone. By day 7, the pirfenidone group achieved a significantly higher epithelialization rate (98.7% ± 1.8%) compared to the control group on day 10 (83.6% ± 14.09%). Histopathological analysis revealed greater epidermal thickness and better-developed granular layers in the pirfenidone group, indicating improved epithelial maturation.

The study concluded that topical pirfenidone gel significantly accelerated epithelialization in skin graft donor sites, with nearly complete wound closure within seven days. Burn wounds that heal quickly are unlikely to develop hypertrophic scarring, as the inflammatory and proliferative phases that contribute to fibrosis are shortened [58,59]. Treating the fresh wounds was painful at first, but became more well-tolerated as healing progressed. While these findings are promising, the authors acknowledge limitations such as the small sample size and recommend larger, multicenter trials to confirm efficacy.

#### 3.2.4. Efficacy and Safety of Pirfenidone in Patients with Second-Degree Burns: A Proof-of-Concept Randomized Controlled Trial

This randomized controlled trial evaluated the efficacy and safety of oral pirfenidone in managing second-degree burn wounds [53]. Eight patients with recent split-thickness burns were randomized to receive standard burn care alone or standard care with oral pirfenidone (600 mg once daily) for 7 days. The primary endpoint was epidermal thickness as a measure of re-epithelialization, and secondary endpoints included histologic markers of fibrosis, basal membrane organization, and collagen formation within the wound. Results showed that patients receiving oral pirfenidone demonstrated significantly increased epidermal thickness on day 7, suggesting accelerated re-epithelialization in the pirfenidone-treated wounds.

The study concluded that oral pirfenidone shows considerable promise in enhancing re-epithelialization and reducing fibrosis in burn wounds, providing a potential therapeutic tool for improving outcomes in burn patients. The trial’s findings suggest that pirfenidone’s anti-fibrotic effects extend to practical outcomes in burn wound management by potentially reducing the development of pathological scarring through modulation of TGF-β1 pathways. Whether this systemic effect would translate to the topical application remains to be tested; however, the proposed mechanism and efficacy of the oral therapy align with the results reported in the topical trials.

### 3.3. Adverse Reactions

Oral PFD is associated with a range of well-documented, dose-dependent side effects [26]. Data from the CAPACITY IPF clinical trials demonstrated a clear dose–response relationship for these side effects. At the higher dose of 2403 mg/day, nausea occurred in 36% of patients, rash in 32%, and photosensitivity in 12%; this contrasts with the lower 1197 mg/day dose, which was associated with reduced rates of nausea (25%), rash (22%), and photosensitivity (9%) [27]. Low doses of oral PFD, such as the 600 mg per day dose used by Mecott et al. for burn patients [53], would offer a better safety profile, although adverse events have been reported at this dose [60]. Topical PFD avoids metabolism in the gastrointestinal tract, and published clinical trials do not report systemic side effects or liver function test abnormalities [32,36,52,53]. However, it is not known to what extent topical PFD reaches the systemic circulation, and the published studies were not powered to detect uncommon adverse events.

The primary adverse event reported in clinical trials of topical PFD was mild, localized skin irritation. For example, in a trial of 8% pirfenidone gel for localized scleroderma, 11 of 12 patients experienced a “slight, short-term burning sensation” at the application site, which was easily managed and did not require treatment discontinuation [36]. A risk of phototoxicity also exists due to the ability of PFD to absorb UV radiation [26,27,60,61]. Topical application, by concentrating the drug in the skin, may heighten this risk. Consequently, stringent sun protection is critical for patients using topical PFD. The recommendations for patients on oral PFD (sun avoidance, wearing protective clothing, and sunscreen) are applicable to those using topical formulations [26,60]. The lack of reported photosensitivity in the small topical PFD trials may be attributable to effective patient counseling on sun avoidance or the limited statistical power of these studies.

## 4. Discussion

Topical pirfenidone shows promise as an anti-fibrotic agent for burns, skin grafts, scleroderma, and scarring. However, the clinical trials are few in number and leave open several important gaps in knowledge and practical considerations. One key aspect of topical therapy requiring further investigation is the precise mechanism of action of pirfenidone in the dermis as opposed to other tissue types. For instance, while pirfenidone suppresses collagen (COL1A1) mRNA expression in lung fibroblasts [14] and myometrial cells [12], this effect is not observed in keloid [45] or dermal fibroblasts [47], suggesting tissue-specific responses to the drug. These differential effects highlight the need for deeper mechanistic studies specifically focused on skin.

Even within the same tissue, there exist unique subpopulations of fibroblasts. Single-cell and spatial transcriptomics have revealed that the skin’s fibroblast population is not uniform but is composed of multiple distinct subtypes, each with specialized roles in either promoting regenerative healing or driving fibrotic scarring [62,63,64]. It is unknown whether pirfenidone exerts its anti-fibrotic effects uniformly across all fibroblast subpopulations or if it selectively targets the specific pro-fibrotic subtypes.

Another area for further testing is to define the optimal delivery media for pirfenidone, which can be combined with other interventions. While various formulations have been studied, including gels, ointments, and adhesive patches, direct comparisons between delivery systems are lacking. Future research should focus on determining the most effective concentration and delivery vehicles to maximize delivery to the dermis and efficacy within the delivery site. The combination of pirfenidone with other topical agents such as antioxidants has shown promise in rodent models [31,35] and is also a needed direction for future trials. PFD is often combined with the tyrosine kinase inhibitor nintedanib in treating IPF, although this combination has not been reported on in the treatment of skin fibrosis.

Future development of PFD may include modifications to the chemical structure itself in addition to optimizing the methods. Medicinal chemists have explored 5-methyl amide replacements to fine-tune lipophilicity and slow metabolism [65], introduced hydroxyl groups to reduce toxicity without sacrificing potency [66], and built NSAID conjugates to add anti-inflammatory power [67]. Nintedanib is an example of an anti-fibrotic that has been successfully modified to improve pharmacokinetics and efficacy [68].

While promising, the current published clinical evidence for topical pirfenidone is limited by several factors. Most studies have small sample sizes (*n* < 35) and relatively short follow-up periods (≤6 months), making it difficult to assess long-term outcomes and safety. The strongest evidence comes from burn scar treatment in pediatric patients, but even these studies would benefit from larger trials with longer follow-up periods. Armendariz-Borunda et al. reported continuing improvement at the end of a six-month trial evaluating an 8% pirfenidone gel, suggesting long-term improvements [32]. However, current published studies do not chronicle enough time to observe a plateau of topical PFD benefits.

Pirfenidone’s therapeutic profile must be considered within the context of established scar treatments that demonstrate well-documented efficacy. Intralesional corticosteroid injection with triamcinolone acetonide is an effective treatment for keloid and hypertrophic scar treatment, demonstrating significant reductions in scar size, vascularity, and pliability compared to untreated controls, though response rates vary between 50 and 100% with recurrence rates of 33% at one year and 50% at five years [69,70]. Notably, in vitro studies reveal that pirfenidone, along with other pleiotropic anti-fibrotic agents, achieved greater collagen type I reduction than triamcinolone acetonide under controlled conditions, suggesting superior anti-fibrotic potential [51]. Silicone gel therapies, widely considered first-line treatment for abnormal scarring, are effective when applied more than four days per week [71,72]. Laser therapies, particularly fractional CO_2_ laser and pulsed dye laser, offer minimally invasive alternatives with low adverse event rates (2.4% in burn scar treatment), though evidence quality remains limited with most studies showing unclear impact on scar severity compared to other treatments [73,74]. While pirfenidone’s multi-modal mechanism targeting TGF-β pathways presents theoretical advantages over these established treatments, the current clinical evidence base for topical pirfenidone remains significantly smaller than that supporting corticosteroids, silicone-based products, and laser therapies.

Practical barriers to pirfenidone use exist in the United States and other countries. Standard oral regimens used for IPF carry a high side effect burden [26]. Clinicians may be reluctant to prescribe oral pirfenidone for skin conditions due to its established adverse effect profile and lack of familiarity with the drug. Despite its potential, topical pirfenidone formulations are not widely commercially available outside of specific markets like Mexico [37,56]. Although the patent on topical and systemic pirfenidone has expired [7], the drug is rarely prescribed, so costs to obtain may be prohibitively high [75]. The lack of standardized compounding formulations further complicates its clinical application, as different pharmacies may produce variations in quality, strength, and consistency of topical preparations.

## 5. Conclusions

Pirfenidone represents a promising topically applied therapeutic option for various dermatologic conditions characterized by excessive scarring and fibrosis. Its multi-modal mechanism of action, including modulation of the TGF-β pathway and anti-inflammatory effects, makes it particularly attractive for treating pathological scarring. While early clinical evidence is encouraging, particularly in burn scars and localized scleroderma, larger clinical trials are needed to establish its role in dermatologic practice definitively. Necessary areas in drug development are optimizing delivery methods, establishing formulations, and conducting larger-scale clinical trials with longer follow-up periods. Addressing these gaps will help reveal pirfenidone’s potential as a valuable addition to topical dermatologic therapies.

## Figures and Tables

**Table 1:** Summary of clinical studies.

Reference	Population	Main Outcomes	Safety
Armendariz-Borunda et al. (2012) [32]	A total of 63 pediatric patients with hypertrophic burn scars were assigned to topical pirfenidone (*n* = 33) or pressure therapy (*n* = 30).	The pirfenidone group showed a 43% improvement on the VSS, compared to a 16% improvement in the control group after six months.	No adverse events were reported.
Rodriguez-Castellanos et al. (2014) [36]	A total of 12 adult patients with active localized scleroderma.	The study showed a decrease in the mLoSSI score (from a mean of 5.83 to 0.83) and histologic improvement after six months of treatment.	A slight, temporary burning sensation after application was reported by 92% of patients. No systemic or laboratory adverse effects were found.
Mecott et al. (2018) [52]	In total, 24 adult patients requiring split-thickness skin grafts received either topical pirfenidone (*n* = 19) or usual care (*n* = 5).	At day 10, the pirfenidone group had a higher rate of epithelialization (99.5% vs. 88.6%) and greater mean epithelial thickness (108 µm vs. 75.1 µm) compared to the control group.	The initial application caused moderate pain that became well-tolerated over time. No systemic or laboratory adverse effects were found.
Mecott et al. (2020) [53]	A total of 8 patients with second-degree burns were randomized to oral pirfenidone (*n* = 5) or usual care (*n* = 3).	At day 7, the pirfenidone group had a thicker re-epithelialized epidermis (148.98 µm vs. 119.27 µm) and less observed fibrosis.	No alterations in liver or renal function panels were found.

## Data Availability

No new data were created or analyzed in this study. Data sharing is not applicable to this article.

## Abbreviations

The following abbreviations are used in this manuscript:

PFD	Pirfenidone
IPF	Idiopathic Pulmonary Fibrosis
TGF-β	Transforming Growth Factor-Beta
